# Preoperative executive functioning impairments in patients with a meningioma: does a frontal location matter?

**DOI:** 10.1007/s11682-024-00886-7

**Published:** 2024-05-09

**Authors:** Paul Beele, Sander M. Boelders, Geert-Jan M. Rutten, Wouter de Baene, Karin Gehring

**Affiliations:** 1 Department of Neurosurgery, Elisabeth-TweeSteden Hospital, Tilburg, The Netherlands; 2 Department of Cognitive Science and Artificial Intelligence, Tilburg University, Tilburg, The Netherlands; 3 Department of Cognitive Neuropsychology, Tilburg University, Tilburg, The Netherlands

**Keywords:** Meningioma, Executive functions, Frontal lobe

## Abstract

Patients with meningiomas frequently exhibit impairments in executive functioning. There are few studies specifically examining the role of frontal meningioma localization in executive functioning impairments. This study examines whether frontally located meningiomas are specifically associated with executive functioning impairments in a large sample of meningioma patients before treatment, using an axis-wise and lobe-based approach to meningioma localization. We retrospectively examined cognitive performances in 353 patients with frontal, frontally-involved and non-frontal meningiomas on a battery of tests including tests of executive functioning. We applied an axis-based approach to meningioma location, in addition to qualitative lobe-based localization. We examined the association between meningioma coordinates on an anterior-posterior axis and continuous cognitive performance scores in univariate correlations and linear regression analyses. We also examined the association between meningioma coordinates on an anterior-posterior axis with cognitive impairments in multivariable logistic regression analyses. Meningioma position on the anterior-posterior axis was only univariately associated with mean performance on the Stroop test Interference ratio and Symbol Digit Coding task. There was no (multivariable) association with impairments on tests of executive or non-executive domains. Increased odds of impairment on executive functioning tasks were associated with left-localization (Verbal Fluency) and larger meningioma volumes (Shifting Attention). We did not find a specific relation between a frontal meningioma location and executive functioning impairments, which may be explained by widespread organization of executive functioning throughout the brain, diffuse cognitive effects of the mass of meningiomas, functional reorganization due to neuroplasticity, or functional involvement of less-anteriorly located frontal areas.

**Supplementary Information:**

The online version contains supplementary material available at 10.1007/s11682-024-00886-7.

## Introduction

Patients with meningiomas frequently exhibit impairments in executive functioning (Meskal et al., 2016). Executive functioning is a cognitive domain that refers to ‘a set of general-purpose control mechanisms, regulating the dynamics of human cognition and action’ (Miyake & Friedman, 2012). Several distinct, yet correlated executive functions are reported in literature, including cognitive flexibility, inhibitory control, working memory, and strategy use (Diamond, 2013; Miyake et al., 2000; Miyake & Friedman, 2012).

Impairments in executive functioning have been negatively associated with health-related quality of life in patients with meningiomas (Haider et al., 2021; Waagemans et al., 2011) and contribute to functional outcomes such as return to work (Sekely et al., 2022). As patients with meningiomas have a favorable long-term survival (Goldbrunner et al., 2021), it is important to provide insight into expected executive functioning (Haider et al., 2021).

Overall, meningiomas affect cognitive functioning through mass effect and peritumoral edema (Ahmeti et al., 2023; Whittle et al., 2004). In clinical practice, patients with frontal meningiomas are often expected to have executive functioning impairments, due to the location of their meningioma (Alexander & Stuss, 2000; Picton et al., 2007; Stuss & Alexander, 2009). However, to what extent a frontal or non-frontal meningioma location specifically affects executive functioning remains largely unanswered.

While various studies have reported on impaired executive functioning components in meningioma patients (Abel et al., 2016; Bommakanti et al., 2016; De Baene et al., 2019; Dijkstra et al., 2009; Hendrix et al., 2017; Kang et al., 2020; Liouta et al., 2016; Meskal et al., 2015, 2016; Rijnen et al., 2019; Tucha et al., 2003; Van Nieuwenhuizen et al., 2007, 2013, 2019; Waagemans et al., 2011), evidence on the association between meningioma location and executive functioning impairments is often hampered by methodological issues. Most prior studies examined executive functioning in small samples (Abel et al., 2016; Bommakanti et al., 2016; Hendrix et al., 2017; Kang et al., 2020; Liouta et al., 2016; Meskal et al., 2015; Tucha et al., 2003; Van Nieuwenhuizen et al., 2007, 2013, 2019), did not explicitly compare executive functioning performances or performances in other domains between frontal and non-frontal meningiomas (Bommakanti et al., 2016; Hendrix et al., 2017; Meskal et al., 2015; Rijnen et al., 2019; Van Nieuwenhuizen et al., 2019), excluded non-frontal meningiomas from analyses (Tucha et al., 2003), separately compared skull base (anterior and middle fossa) and convexity (anterior and posterior) meningiomas (Liouta et al., 2016), or compared executive functioning performances in specific frontal substrates to the rest of the brain (Abel et al., 2016). Furthermore, most studies applied rough anatomical labels to relate meningioma locations to the occurrence of executive impairments (Bommakanti et al., 2016; Hendrix et al., 2017; Kang et al., 2020; Liouta et al., 2016; Meskal et al., 2015; Rijnen et al., 2019; Tucha et al., 2003; Van Nieuwenhuizen et al., 2019).

The present study aimed to examine the relation between executive functioning performance and meningioma location in a large sample of pretreatment patients, focusing on location along the anterior-posterior axis. We hypothesized that executive functioning and deficits thereof are not exclusively related to a more anterior meningioma location. To quantify the location of the meningioma we applied an axis-based coordinate approach, in addition to a lobe-based localization.

## Materials and methods

### Design

This study was conducted as part of a prospective longitudinal study on cognitive outcomes in brain tumor patients admitted for surgical resection between November 2010 and November 2019 at the Elisabeth-TweeSteden hospital (Tilburg, the Netherlands). Patients underwent a neuropsychological screening (NPS) one to four days prior to surgery.

### Sample

Data from adult patients with a WHO grade I or grade II meningioma who completed the pre-surgical cognitive assessment were used in the current study. Grade III meningiomas were excluded because of infiltrative growth characteristics (Louis et al., 2016). The sample used in the current study has certain overlap with patient samples from previous studies (De Baene et al., 2019; Meskal et al., 2015; Rijnen et al., 2019; Van Lonkhuizen et al., 2019). Supplementary Fig. 1 shows a flowchart of patient inclusion. Patients with multiple meningiomas were excluded, as the effect of individual meningiomas could not be reliably assessed.

## Measures and procedures

### Patient and clinical characteristics

Patients provided sociodemographic information such as age, sex, and years of education classified according to The Dutch Verhage scale (Verhage, 1964). A histopathological diagnosis was categorized according to the WHO classification of central nervous system tumors (Louis et al., 2016). We recorded the use of psychotropic medication. We collected the American Society of Anesthesiologist (ASA) score as a measure of physical health status with patients considered healthy (ASA scores I-II) or having substantial comorbidities (ASA scores III-IV) (Dripps, 1963). The Dutch translation of the Hospital Anxiety and Depression Scale (HADS) was used to assess anxiety and depression symptoms (Spinhoven et al., 1997; Zigmond & Snaith, 1983).

### Location characteristics

We determined meningioma location based on gadolinium-enhanced T1-weighted MRI scans collected one day before surgery, using a Philips Achieva 3T MRI-scanner (Philips Medical Systems, Best, The Netherlands). Voxel size varied between 0.68 × 0.68 × 0.8 mm and 1 × 1 × 1 mm (median: 0.8 × 0.8 × 0.8 mm). Preoperative tumor volume was determined on semi-automatically segmented lesions using ITK-SNAP (www.itksnap.org) (Yushkevich et al., 2006) or BrainLab software (BrainLab, Munich, Germany).

Tumor location was classified in two different ways. Firstly, segmentations were used to assign coordinates to the tumor. T1 scans and their corresponding segmentations were normalized to MNI space using affine transformation (code available at http://cmictig.cs.ucl.ac.uk/wiki/index.php/Reg_aladin) (Ourselin et al., 2001). After normalization, voxel sizes were isotropic at 1 × 1 × 1 mm. Tumor location was defined as the center of the mass of segmentation in MNI space (Fonov et al., 2011). Coordinates were assigned along three axes to quantify meningioma locations: frontal location from posterior to anterior (range: 0-233 millimeters), lateral location from left to right (range: 0-197 millimeters), and height from caudal to cranial (range: 0-189 millimeters).

Secondly, we classified meningiomas either as non-frontal (e.g. temporal, parietal, occipital), frontally involved (e.g. frontal-parietal) or solely frontal to reproduce previous research on meningioma location and executive functioning.

### Cognitive performance

Patients completed the Dutch translation of the computerized battery Central Nervous System Vital Signs (CNS VS) (Gualtieri & Johnson, 2006). This study analyzed seven of the neuropsychological test variables as measured with this test battery (Supplementary Table 1). In addition, patients performed the non-computerized Dutch version of the Controlled Oral Word Association letter fluency test (verbal (phonemic) fluency) (Schmand et al., 2008). Starting in 2015, we additionally admitted the WAIS Digit Span task (Forward and Backward subtests) (Wechsler, 2008).

Raw test scores were converted into sociodemographic adjusted *z* scores based on Dutch normative data (Rijnen et al., 2020). *Z* scores for the letter fluency test were calculated by correcting for educational levels (Schmand et al., 2008). Digit span scores were corrected for age, sex, and educational level, using data from another Dutch control group (from CAR Study A, ClinicalTrials.gov reference NCT02953756). For all tests, higher *z* scores indicate better performances. Standardized *z* scores were also dichotomized into impaired (*z* score ≤-1.5) or non-impaired (*z* score >-1.5) performances (Lezak & Howieson, 2012).

Cognitive tests were divided into two groups, based on their reliance on executive functioning (Supplementary Table 1). We categorized the Shifting Attention test (cognitive flexibility), Stroop test Interference ratio (inhibitory control), Verbal Fluency task (strategy use), and Digit Span Backward test (working memory) as primary executive functioning tasks (Diamond, 2013).

### Statistical analyses

We compared the proportions of solely frontal, frontally involved, and non-frontal meningiomas between included and excluded patients, and between patients who did or did not complete a cognitive task through chi-square tests of independence. We compared patient characteristics between meningioma location samples through one-way ANOVA tests, Kruskal-Wallis tests, and chi-square tests of independence as appropriate. Cognitive test scores of patients with meningiomas were compared to those of normative controls for every subgroup based on frontal lobe involvement.

### Axis-based coordinate associations with mean cognitive performance and impairments

We examined the associations and linearity between meningioma coordinates on the anterior-posterior axis and *mean cognitive performance* in terms of *z* scores on each cognitive test through correlations and univariate linear regression analyses. Multivariable linear regression analyses were not conducted if the assumption of linearity was not met (i.e. Pearson’s *r* < .30) (Field, 2013). Covariates for multivariable analyses were based on previous literature (Meskal et al., 2016; Taphoorn & Klein, 2004), including meningioma location on the left-right axis and caudocranial axis, volume, age, sex, educational level, WHO meningioma grade (Louis et al., 2016), ASA grade (comorbidity), HADS anxiety and depression scores.

We examined the percentage of patients with *impairment on each cognitive test* for the patient subgroups with different frontal lobe involvements. We performed multivariable logistic regression analyses to evaluate the predictive value of the continuous meningioma anterior-posterior coordinates on the odds of executive functioning test impairments and in tests that assess executive functioning to a lesser extent (Box & Tidwell, 1962; Field, 2013; Stevens & Pituch, 2016; Tukey, 1977). We included main effects and interaction effects between coordinates on the anterior-posterior axis with coordinates on the left-right axis, coordinates on the caudal-cranial axis, and tumor volume. In addition, we included covariates for multivariable analyses as mentioned above (Meskal et al., 2016; Taphoorn & Klein, 2004). Meningioma coordinates and volume were centered around their means to avoid multicollinearity with interaction effects (Aiken & West, 1991).

### Associations of lobe-based classifications with cognitive impairment

To reproduce previous research on meningioma location and executive and non-executive functioning *impairments*, we repeated our multivariable analyses with an anatomical classification based on lobe involvement, instead of an axis-based approach. The Benjamini-Hochberg procedure was used to set *p* values of our multivariable logistic regression analyses against a multiple-testing corrected significance level, with a 5% false discovery rate (Benjamini & Hochberg, 1995).

### Brain maps of distributions of cognitive impairments

Finally, we created brain maps to graphically depict the distribution of cognitive impairments in relation to meningioma location. To this end, we computed voxel-wise maps for all meningioma patients. For the impairment probability per voxel, we calculated the percentages of patients with impaired task performances given the presence of a meningioma at that particular voxel. Supplementary File 1 provides calculation examples. The Python code for this calculation is available at https://github.com/ETZ-TZO/visualize-cognitive-function.

All analyses were performed using SPSS version 28.0 (IBM, Armonk, New York, USA).

## Results

### Patient characteristics

The proportions of solely frontal, frontally involved, and non-frontal meningioma subgroups in this study did not differ significantly from excluded patients who were unable to undergo NPS (*N* = 37) (χ^2^(2) = 3.80, *p* = .150). Test scores of included patients were occasionally missing or invalid, for example due to being too tired or performing tests incorrectly despite instructions. The proportion of patients with frontal, frontally involved, or non-frontal meningiomas in this sample did not differ between patients who completed a test and those who did not (all *p*’s > 0.05).

**Fig. 1:**
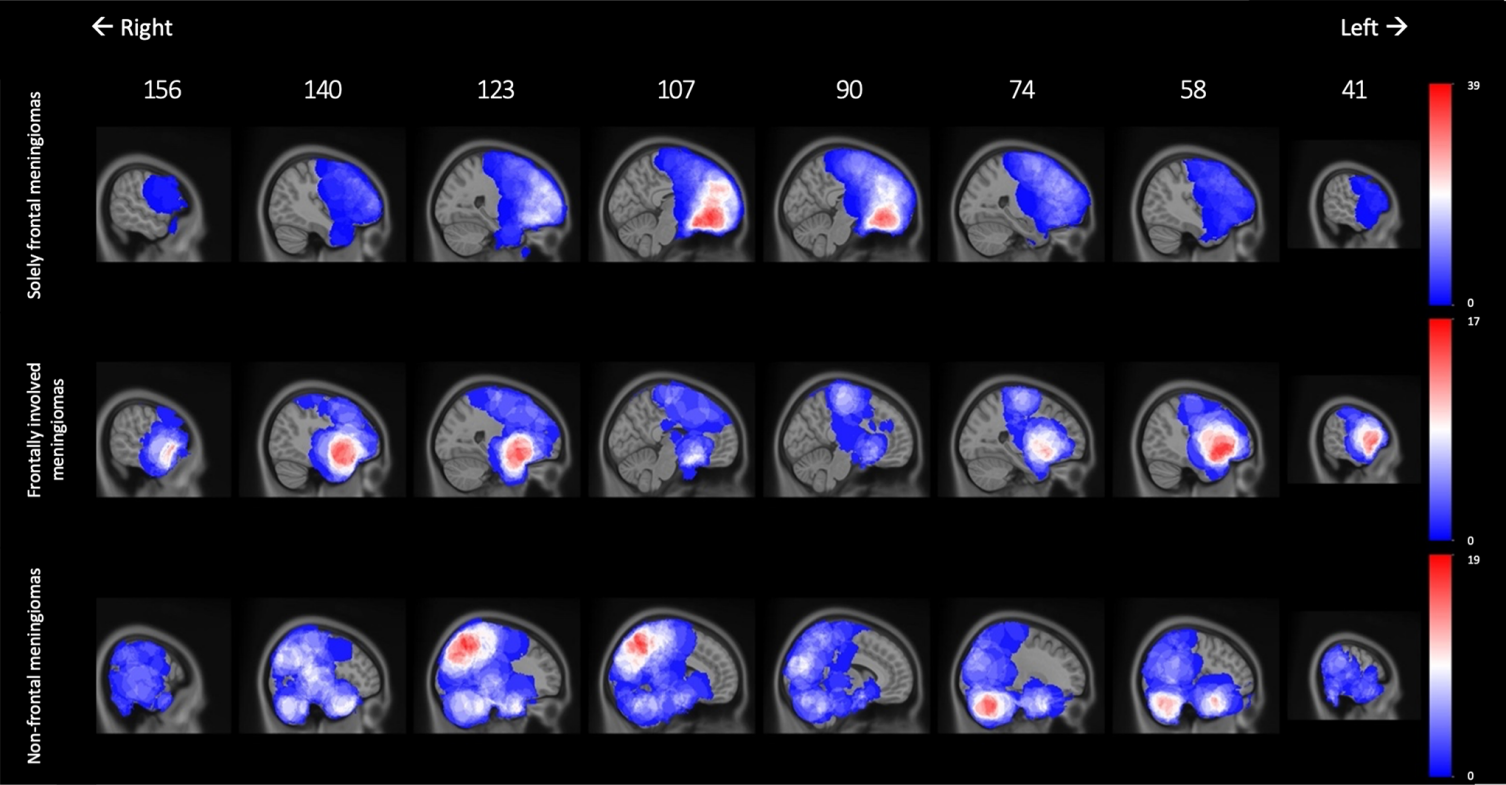
Meningioma localization maps, indicating the number of meningiomas in the dataset involved within each voxel for solely frontal meningiomas (top, range 0–39), frontally involved meningiomas (middle, range 0–17), and non-frontal meningiomas (bottom, range 0–19) in sagittal views from right to left. Sagittal plane ranges from 0 (left hemisphere) to 197 (right hemisphere). Red color indicates the presence of more meningiomas per voxel

**Fig. 2:**
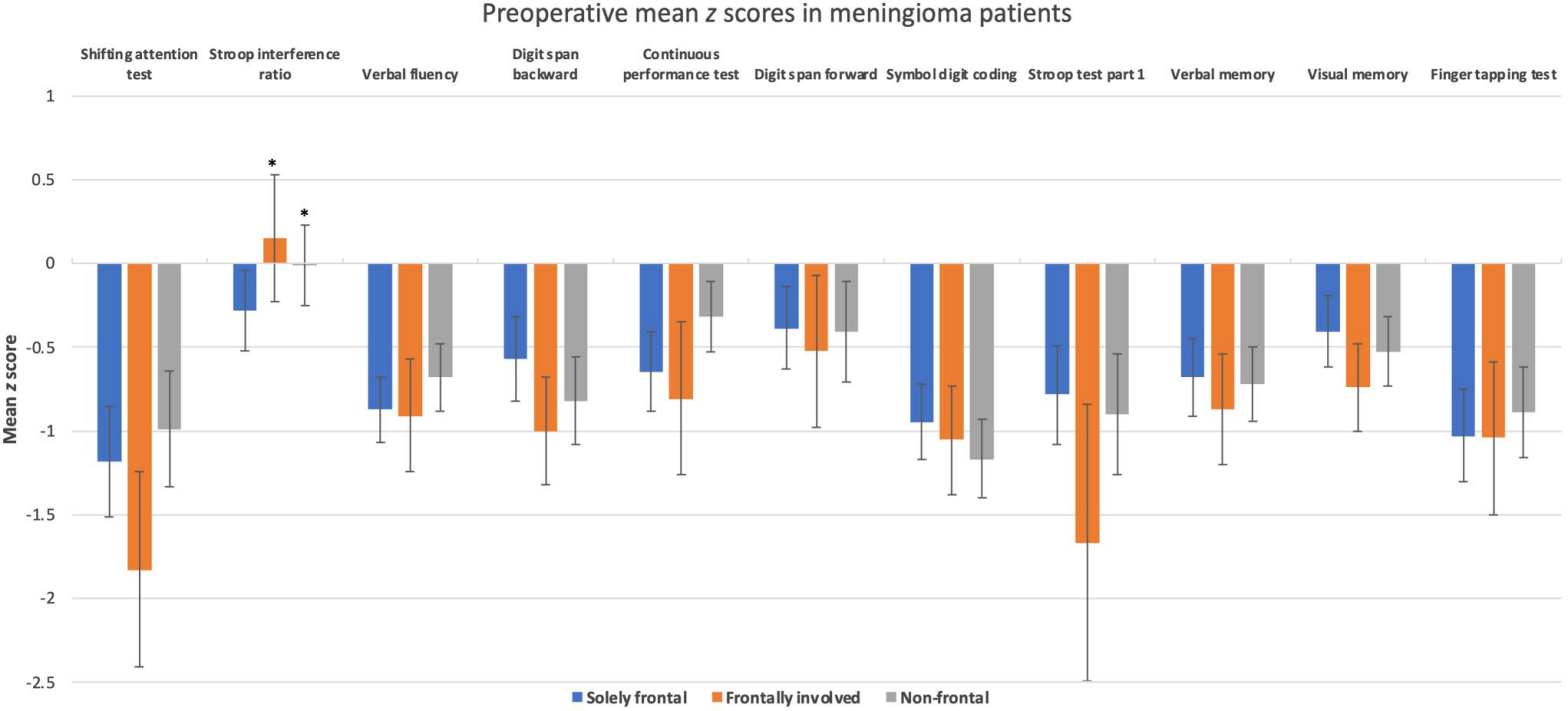
Preoperative mean z scores in solely frontal (blue), frontally involved (orange), and non-frontal meningioma patients (grey). Non-significant differences from the normative sample (z score = 0) are marked by the asterisk. Error bars represent 95% confidence intervals

Table 1 presents the characteristics of the solely frontal, frontally involved, and non-frontal meningioma samples. In total, 353 meningioma patients were included (solely frontal *N* = 148, 41.9%; frontally involved *N* = 64, 18.1%; non-frontal *N* = 141, 39.9%). Figure 1 illustrates the distribution of meningioma locations. Patients with meningiomas had worse mean performances compared to normative controls on almost all cognitive tests across meningioma locations (Fig. 2).

**Table 1:** Baseline characteristics of meningioma patients with frontal or non-frontal meningiomas

Characteristics	Solely Frontal (*N* = 148)	Frontally Involved (*N* = 64)	Non-frontal (*N* = 141)	χ ^2^/ANOVA/KW test^a^ (*p* value)^a^
	Mean ± SD (range); *n* (%); median (IQR)	Mean ± SD (range); *n* (%); median (IQR)	Mean ± SD (range); *n* (%); median (IQR)	
Age (years)	58.1 ± 11.7 (23.0–84.5)	55.7 ± 12.4 (46.0–65.5)	57.3 ± 12.1 (29.0–82.0)	0.900 (0.407)
Sex				2.313 (0.315)
Male	45 (30)	13 (20)	40 (28)	
Female	103 (70)	51 (80)	101 (72)	
Educational level^b^				10.041 (0.040)*
Low	62 (42)	18 (28)	53 (38)	
Middle	39 (26)	14 (22)	46 (33)	
High	47 (32)	32 (50)*	42 (30)	
WHO Grade				2.254 (0.324)
Grade I	134 (91)	61 (95)	133 (94)	
Grade II	14 (9)	3 (5)	8 (6)	
Tumor volume (cm^3^)^c^	33.8 (16.9–67.8)	54.0 (25.5–95.8)*	40.7 (20.6–70.2)	7.855 (0.020)*
Anterior-posterior axis (mm)^c, d^	161.7 (145.0–175.4)*	143.3 (123.7–150.1)*	93.7 (72.0–124.0)*	-184.335 (< 0.001)*
Left to right axis (mm)^c, d^	99.6 (87.4–110.9)	91.3 (61.7–130.5)	109.6 (69.0–125.8)	1.110 (0.574)
Caudo-cranial axis (mm)^c, d^	79.4 (58.6–111.8)	73.6 (59.3–117.1)	55.9 (42.2–102.4)*	-19.371 (< 0.001)*
ASA score				5.971 (0.051)
I-II	102 (79)	46 (90)	108 (89)	
III-IV	27 (21)	5 (10)	13 (11)	
Psychotropic medication				0.145 (0.930)
Yes	69 (48)	31 (50)	69 (50)	
No	75 (52)	31 (50)	69 (50)	
Presentation with epilepsy				3.040 (0.219)
Yes	25 (21)	7 (14)	30 (26)	
No	95 (79)	42 (86)	84 (74)	
HADS anxiety^e^				1.684 (0.431)
≥ 8 points	55 (37)	29 (45)	51 (36)	
< 8 points	93 (63)	35 (55)	90 (64)	
HADS depression^e^				1.791 (0.408)
≥ 8 points	40 (27)	23 (36)	40 (28)	
< 8 points	108 (73)	41 (64)	101 (72)	

The number of patients and measures differ over variables due to missing or invalid scores. ^a^Chi-square test, ANOVA F test, and Kruskal-Wallis *H* test values. Statistical significance was considered as *p* < .05, marked by the asterisk in bold. ^b^Grouped based on the Dutch Verhage scale; significant contributor assessed through post-hoc testing. ^c^Data were assessed through non-parametric Kruskal-Wallis *H* tests because of outliers and skewness of data. ^d^Higher values indicate meningiomas located more frontally, more to the right, and more cranial. ^e^HADS, Hospital Anxiety and Depression Scale

### Axis-based coordinates and mean cognitive performance

Univariate correlation and linear regression analyses indicated that more anterior meningioma coordinates on the anterior-posterior axis correlated slightly with lower *z* scores on the Stroop interference ratio (*r*(304)=-0.118, *p* = .019) and were slightly associated with higher *z* scores on the Symbol Digit Coding task (*r*(318) = 0.113, *p* = .022) (Supplementary Table 2, Supplementary Fig. 2). Multivariable analyses were not performed, as the assumption of linearity was not met (Pearson’s *r*s < 0.30).

### Axis-based coordinates and cognitive impairments

**Fig. 3:**
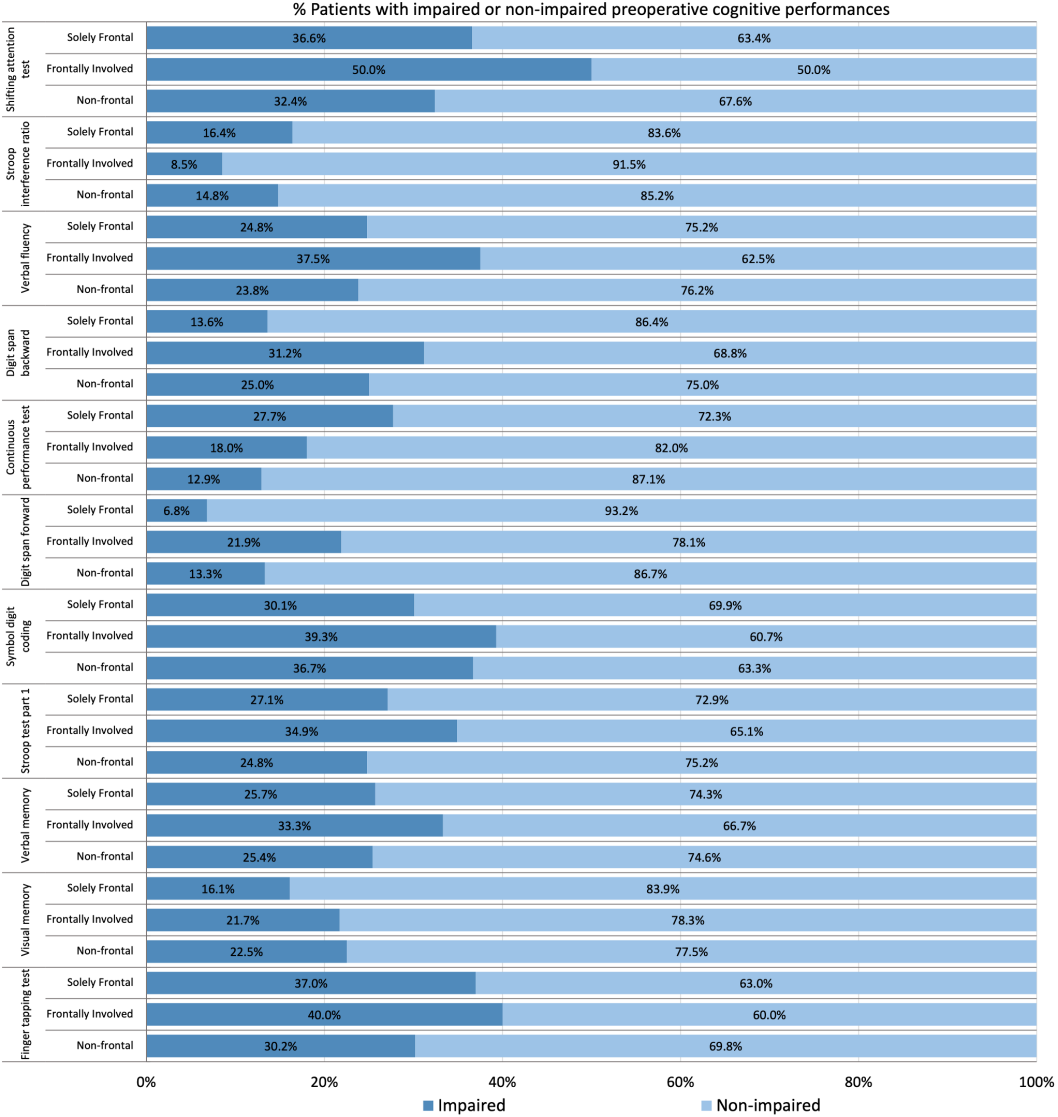
Percentages of patients with preoperative impaired or non-impaired performances on cognitive tasks. Percentages are displayed separately for patients with solely frontal, frontally involved, and non-frontal meningiomas

Cognitive impairments on tests occurred frequently among all meningioma location subgroups (Fig. 3). Supplementary Fig. 3 illustrates the cognitive impairment probability maps per voxel. HADS and ASA scores were not included as covariates in the multivariable logistic regression analyses of the Digit Span Backward and Forward tasks, as inclusion of these variables led to inflated standard errors and decreased interpretability of the multivariable regression analyses. This was the result of a limited number of patients with impairments on the Digit Span tasks (Backward n_impaired_/n_total_ = 32/135; Forward n_impaired_/n_total_ = 18/135) due to later addition of this test to our battery, missing data on the HADS and ASA scores, and poor associations in univariable logistic regression analyses. Meningioma coordinates and other covariates were still included in the multivariable regression model of the Digit Span tasks.

**Fig. 4:**
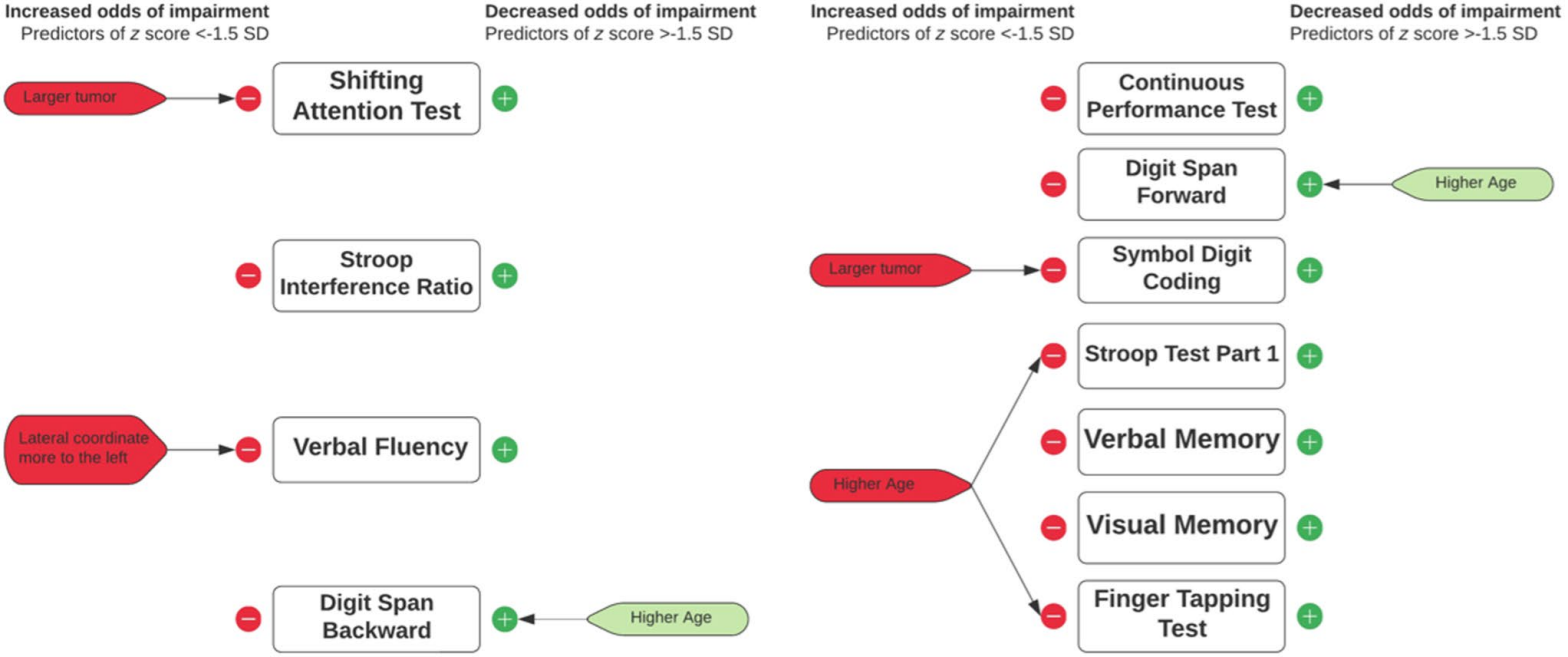
Summary of significant results of multivariable logistic regression analyses with coordinate-based anatomical labels. **Left side**: significant determinants of impairment odds on tests of executive functioning. **Right side**: significant determinants of impairment odds on tests that assess executive functioning to a lesser extent. Predictors of increased impairment odds (z score ≤-1.5 SD) are noted in *red*. Predictors of decreased impairment odds (z score >-1.5 SD) are noted in *green*. Alpha was adjusted using the Benjamini-Hochberg procedure

The meningioma coordinate on the anterior-posterior axis was not significantly associated with odds of impaired performances on any cognitive test (Fig. 4; Supplementary Table 3). No significant interaction effects were found between a meningioma coordinate on the anterior-posterior axis with coordinates on the left-right axis, caudocranial axis, or tumor volume (all *p*’s > Benjamini-Hochberg adjusted alpha).

Instead, these multivariable logistic regression analyses demonstrated several other independent predictors of impairment on various cognitive tests (Fig. 4, Supplementary Table 3). In primary tasks of executive functioning, patients with larger tumors had larger odds of impairment on the Shifting Attention test. A meningioma coordinate more to the left was associated with larger odds on the Verbal Fluency task. Regarding remaining cognitive tests, larger tumors were associated with larger odds of impairment on the Symbol Digit Coding test. A higher age was associated with larger odds of impairment on part 1 of the Stroop test, as well as with larger odds of impairment on the Finger Tapping test. Remarkably, for both Digit Span tasks higher age was associated with smaller odds of impairment, although analyses were performed in limited sample sizes. Repeated Digit Span analyses excluding age as a covariate demonstrated no other independent predictors of impairment (Supplementary Table 4).

### Associations of lobe-based classifications with cognitive impairment

Similar analyses with a lobe-based approach to meningioma location yielded comparable results as the axis-based analyses (Supplementary Fig. 4, Supplementary Table 5) of predictions of cognitive impairments.

## Discussion

Prior studies often concluded that the detrimental effect of a meningioma on executive functioning performance was related in particular to a frontal meningioma location (Abel et al., 2016; Bommakanti et al., 2016; De Baene et al., 2019; Dijkstra et al., 2009; Hendrix et al., 2017; Liouta et al., 2016; Meskal et al., 2015, 2016; Rijnen et al., 2019; Tucha et al., 2003; Van Nieuwenhuizen et al., 2007, 2013, 2019; Waagemans et al., 2011). Due to the nature of these studies, the question whether executive functioning disorders are exclusively related to a (more) frontal meningioma location has not been sufficiently answered. This study sought to answer to what extent the location of a more frontal meningioma location was related to performances and impairments on executive and non-executive functioning tests, using an axis-wise approach to meningioma locations.

We could not demonstrate clear associations between executive functioning (impairments) and the location of a meningioma on the anterior-posterior axis. In univariate analyses, we found significant but small associations between the anterior-posterior coordinates and performances on both an executive and a non-executive cognitive task: a more frontal coordinate was correlated with a (lower) mean performance on the Stroop interference ratio (inhibitory executive control) and with a (higher) mean score on the Symbol Digit Coding task (non-executive information processing). However, multivariable analyses correcting for potential confounders could not be performed for mean cognitive performance scores due to the small correlations in univariate analyses. Odds of impairment on neither executive functioning tasks, nor non-executive tests, were associated with anterior coordinates in this study. Comparable associations were found for the lobe-based classifications with executive and non-executive cognitive performances.

We propose several reasons why the current study did not demonstrate a clear association between a more frontal meningioma location and executive functioning impairments. First, executive functioning is thought to rely on a widespread functional network of subcortical and white matter tracts across different lobes and hemispheres (Duffau, 2012, 2017; Hart et al., 2016). From this perspective, meningiomas cause executive functioning impairments through disruption of a widespread network, instead of causing a local effect. Second, the mass effect through which meningiomas affect cognitive functioning (Whittle et al., 2004) might cause a more diffuse disruption throughout the brain than local infiltration. This may impair cerebral structures necessary for executive functioning, located more distantly from the primary meningioma. This way, the effect of a meningioma on cognitive functioning may not fully depend on its primary location. Third, meningiomas have a slow rate of growth, leaving room for neuroplastic processes (Duffau, 2006). Regardless of whether executive functioning is localized in a specific area in the frontal lobe or organized through widespread functional networks, slow meningioma growth may allow for reorganization of structures necessary for executive functioning. This way, location-associated effects on executive functioning may be mitigated in meningioma patients, compared to patients with acute-onset lesions.

Finally, in literature, it is assumed that a more frontal meningioma is more detrimental to executive functioning, as more anterior brain regions become increasingly responsible for more higher-order cognitive functioning (Koechlin et al., 2003). Yet, for some executive functioning tasks (e.g. set-shifting) it is theorized that they rely on frontal lobe structures, albeit not the most prefrontal areas (Smith et al., 2004). If this were the case, our linear axis-based analyses may not have detected significant location effects on specific aspects of executive functioning. Interestingly, a prior study in a sample partially overlapping with the current patient sample identified the left middle frontal gyrus and the left superior frontal gyrus as important areas for aspects of executive functioning (De Baene et al., 2019). This implies that executive functioning is associated with less-anteriorly located areas of the frontal lobe, instead of the utmost frontal areas or the frontal lobe per se.

In multivariable analyses, we found that shifting attention *impairments* in meningioma patients were independently associated with larger meningioma volumes. For the non-executive cognitive tests, larger meningioma volume was independently associated with more impairments on the Symbol Digit Coding task. We also demonstrated an association between higher age and impairments on response-time-based tasks, which is in line with the concept of decreased processing speed in time-based response tasks in older age (Murman, 2015). These effects were found after correcting patients’ performance scores for (among others) age effects based on healthy controls, which suggest a larger negative effect of aging in patients with meningiomas. Finally, we found that Verbal Fluency impairments were associated with meningiomas located more to the left, in line with the concept of left-sided language lateralization (Knecht et al., 2000).

Less expectedly, we found that higher age was associated with smaller odds of impairment on both Digit Span tasks, contrary to existing literature (Karakaş et al., 2002). This finding may result from the manner in which we constructed *z* scores. The *expected* performance on the Digit Span tasks decreased linearly with age, while it becomes increasingly difficult to reach higher scores (i.e. it is harder to move from ten to eleven correct series compared to moving from one to two correct series). This way, younger patients had to perform excessively better to match their expected performances and maintain a similar *z* score to older patients. Still, neither our primary analyses, nor repeated analyses excluding age as a covariate identified meningioma location as a significant predictor of Digit Span impairment on either subtask.

We acknowledge several limitations with the current study. Our research primarily included patients who were fit for surgery and cognitively apt for preoperative NPS. This may have caused an overestimation of cognitive performance in our study compared to the general meningioma patients admitted for surgery. However, the proportion of frontal meningiomas in the excluded sample did not differ significantly from the analyzed sample. Secondly, we did not measure peritumoral edema. Edema in meningiomas is associated with VEGF release, that induces pathological angiogenesis (Ahmeti et al., 2023; Hou et al., 2013). Edema has been shown to impair cognitive performances in patients with meningiomas (Ahmeti et al., 2023; Bommakanti et al., 2016; van Nieuwenhuizen et al., 2019). Although prior research did not find substantial or significant differences in peritumoral edema volume across different meningioma locations (Frati et al., 2022; Gurkanlar et al., 2005), edema volumes and their effect on cognitive functioning could have varied in our study population.

The strengths of the current study are its large meningioma sample and use of both an axis-based quantification and lobe-based approach to meningioma location. Future studies should further examine the role of specific cerebral areas and their role in executive functioning, from a network-based perspective.

## Conclusion

In conclusion, we found a significant but small, univariable association between a more frontal meningioma location and lower inhibitory control performances, as well as an association with higher information processing performances. However, we demonstrated that meningioma location on the anterior-posterior axis was not associated with larger odds of impairments on tests of executive functioning, nor any cognitive test in the current study. Hypothetically, this may be explained by widespread organization of executive functioning throughout the brain, diffuse mass effect of meningiomas, functional reorganization due to neuroplasticity, or functional involvement of less-anteriorly located frontal areas. Factors such as volume, age, and meningioma location on the left-right axis were independent predictors of impairments on various cognitive tests.

## Electronic supplementary material

Below is the link to the electronic supplementary material.

Supplementary Material 1

Supplementary Material 2: **Supplementary Figure 1**: Flowchart of patient inclusion. NPA; neuropsychological assessment. **Supplementary Figure 2**: Scatterplots illustrating the correlation between the meningioma coordinate on the anterior-posterior axis and z scores on each cognitive test. Higher z scores indicate better performances. **Supplementary Figure 3**: Cognitive impairment visualizations per voxel for meningioma patients. Sagittal plane cross-sections from right to left. Red colors indicate higher impairment probabilities. Cognitive impairments per voxel are calculated as the percentage of impaired meningiomas per voxel. Sagittal plane ranges from 0 (left hemisphere) to 197 (right hemisphere). Impairments range from 100% impairment (red) to 0% impairment (blue) per voxel. **Supplementary Figure 4**: Summary of significant results of multivariable logistic regression analyses with lobe based anatomical labels. Left side: significant determinants of impairment odds on tests of executive functioning. Right side: significant determinants of impairment odds on tests that assess executive functioning to a lesser extent. Predictors of higher impairment odds (z score ≤–1.5 SD) are noted in red. Predictors of lower impairment odds (z score >–1.5 SD) are noted in green. Alpha was adjusted using the Benjamini-Hochberg procedure

Supplementary Material 3

## Data Availability

Stored in institutional repository, available upon request.

## Abbreviations

NPS: Neuropsychological Screening
ASA: American School of Anesthesiologists
CNS VS: Central Nervous System Vital Signs
HADS: Hospital Anxiety and Depression Scale

## Code availability

Available upon request.
