# Supplementary figures and images for: Preoperative executive functioning impairments in patients with a meningioma: does a frontal location matter?
Source: Brain Imaging Behav. 2024 May 9;18(5):989–1000. doi: 10.1007/s11682-024-00886-7 (PMC11582180; doi:10.1007/s11682-024-00886-7)

**Supplementary Figure 1**


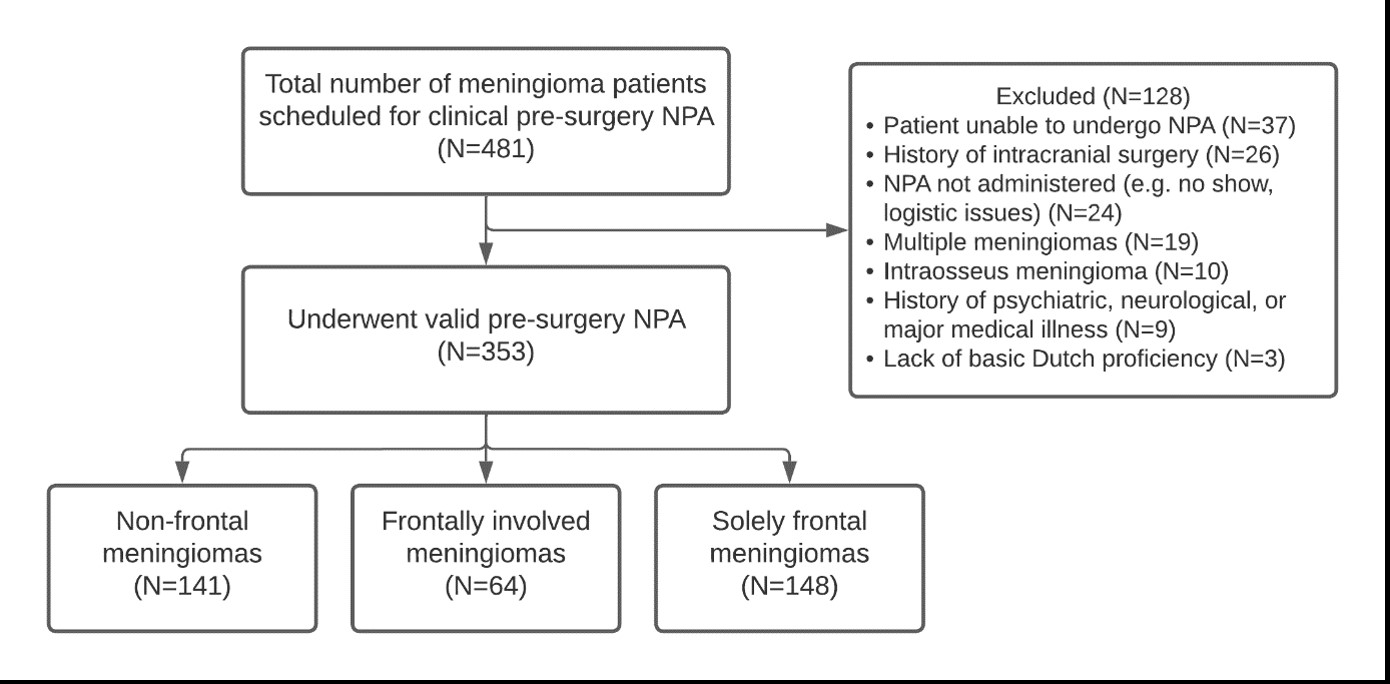


**Supplementary Figure 2**


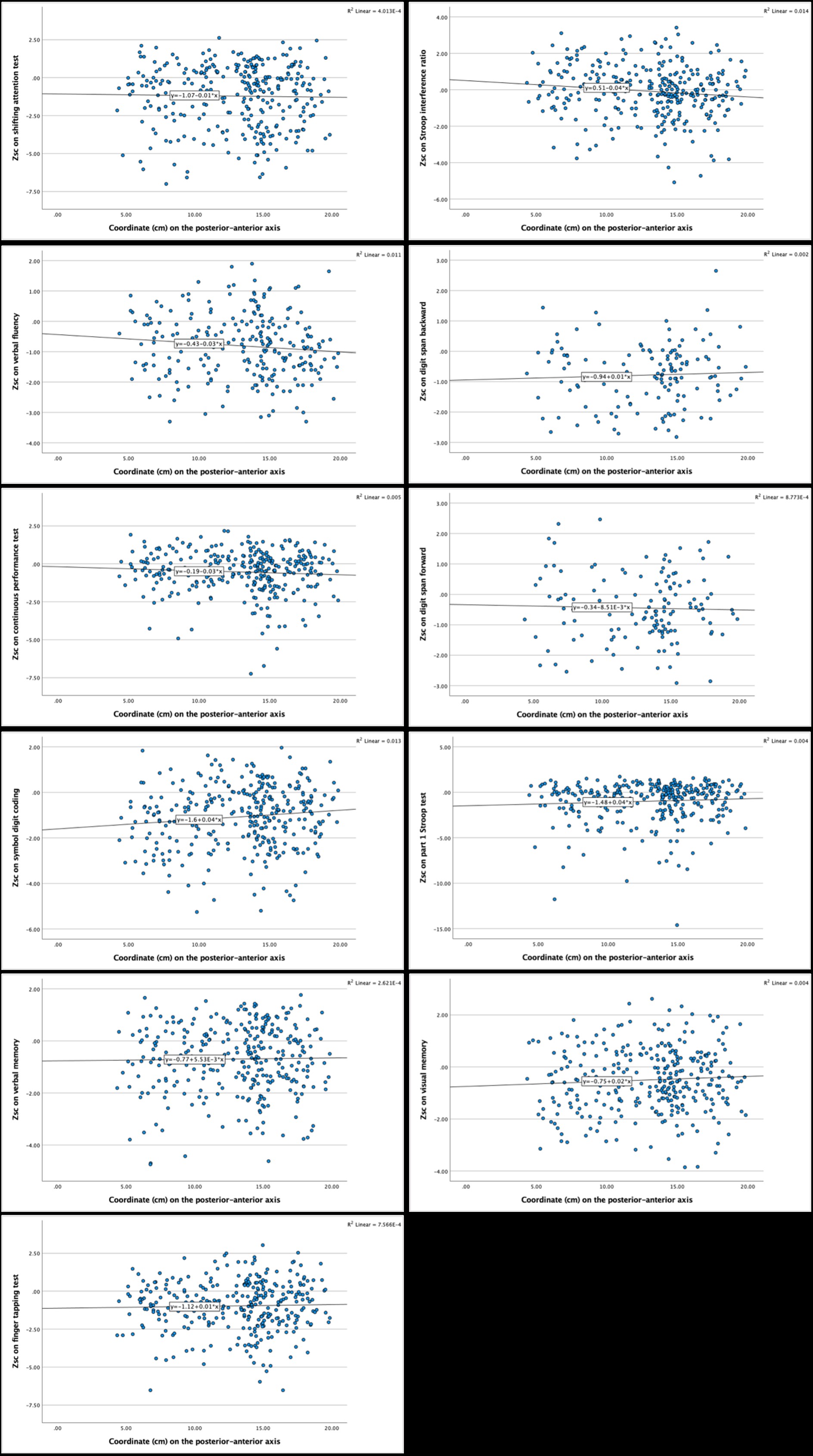


**Supplementary Figure 3**


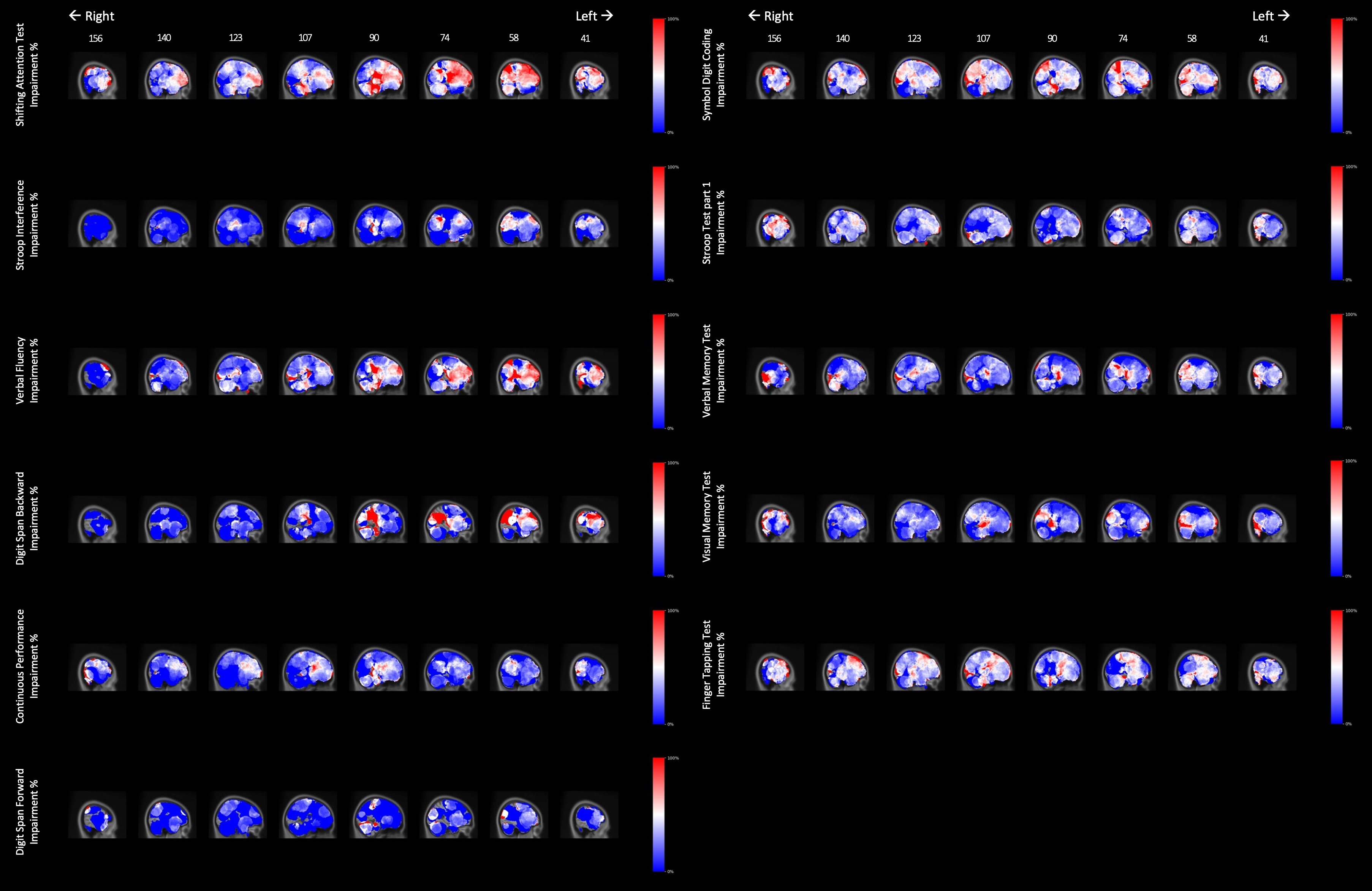


**Supplementary Figure 4**


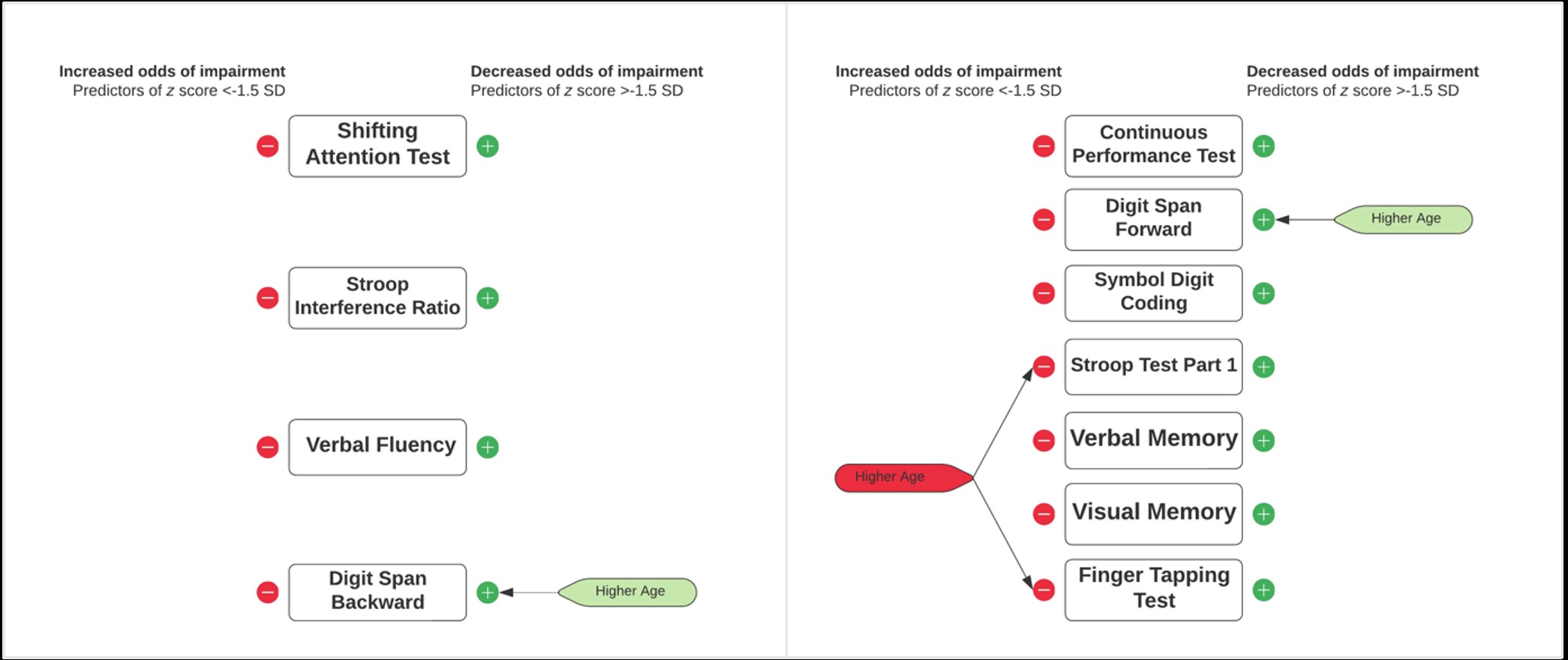

Supplement: Supplementary file 2 — Supplementary Material 2: Supplementary Figure 1: Flowchart of patient inclusion. NPA; neuropsychological assessment. Supplementary Figure 2: Scatterplots illustrating the correlation between the meningioma coordinate on the anterior-posterior axis and z scores on each cognitive test. Higher z scores indicate better performances. Supplementary Figure 3: Cognitive impairment visualizations per voxel for meningioma patients. Sagittal plane cross-sections from right to left. Red colors indicate higher impairment probabilities. Cognitive impairments per voxel are calculated as the percentage of impaired meningiomas per voxel. Sagittal plane ranges from 0 (left hemisphere) to 197 (right hemisphere). Impairments range from 100% impairment (red) to 0% impairment (blue) per voxel. Supplementary Figure 4: Summary of significant results of multivariable logistic regression analyses with lobe based anatomical labels. Left side: significant determinants of impairment odds on tests of executive functioning. Right side: significant determinants of impairment odds on tests that assess executive functioning to a lesser extent. Predictors of higher impairment odds (z score ≤–1.5 SD) are noted in red. Predictors of lower impairment odds (z score >–1.5 SD) are noted in green. Alpha was adjusted using the Benjamini-Hochberg procedure [file 11682_2024_886_MOESM2_ESM.docx]
