# Supplementary material for: Preoperative executive functioning impairments in patients with a meningioma: does a frontal location matter?
Source: Brain Imaging Behav. 2024 May 9;18(5):989–1000. doi: 10.1007/s11682-024-00886-7 (PMC11582180; doi:10.1007/s11682-024-00886-7)
Supplement: Supplementary file 3 — Supplementary Material 3 [file 11682_2024_886_MOESM3_ESM.docx]

# Supplementary

## Supplementary Table 1

| Supplementary Table 1: Description of neuropsychological tests | | | |
| --- | --- | --- | --- |
| **Cognitive Test** | **Description** | **Test score calculation** | **Cognitive aspect tested** |
| *Primary tests of executive functioning* | | | |
| Shifting Attention Test (SAT) | Subject matches geometric objects by shape or color for two minutes. | SAT correct responses – SAT errors. | Cognitive flexibility |
| Stroop Test (ST) Interference ratio | Ratio of ST part 3 and ST part 1 reaction times | (Reaction time ST part 3 – reaction time ST part 1) / reaction time ST part 1. | Inhibitory control |
| Verbal Fluency Test (VF)_a_ | Subject names words starting with a specific letter for 1 minute (3 trials total; D A T). | Total words correct for 3 trials. | Strategy use |
| Digit Span Backward (DSBW)_a_ | Listen to sequences of digits orally and to repeat them as heard in reverse order. Two trials are conducted up to 8 digits per trial. | Total number of digits repeated correctly for 2 trials. | Working memory |
| *Tests that assess executive functioning to a lesser extent* | | | |
| Continuous Performance Test (CPT) | Subject responds to target stimulus (“B”) among distractors for 5 minutes. | Average reaction time of responses to “B”. | Sustained attention |
| Digit Span Forward (DSFW)_a_ | Listen to sequences of digits orally and to repeat them as heard in order. Two trials are conducted up to 8 digits per trial. | Total number of digits repeated correctly for 2 trials. | Sustained attention |
| Symbol Digit Coding (SDC) | Participant matches numbers with symbols for two minutes. | SDC correct responses – SDC incorrect responses. | Information processing |
| Stroop Test (ST) part 1 | Subject presses the space bar as soon as the words RED, YELLOW, BLUE and GREEN appear on screen. | Average ST simple reaction time. | Reaction time |
| Verbal Memory Test (VEM) | Fifteen words presented one at a time. Subject subsequently identifies presented words among new words. After six more tests, there is a delayed recognition memory trial. | VEM direct correct hits + VEM direct correct passes + VEM delayed correct hits + VEM delayed correct passes. | Verbal memory (direct and delayed) |
| Visual memory Test (VIM) | Fifteen abstract images presented one at a time. Subject subsequently identifies presented images among new images. After six more tests, there is a delayed recognition memory trial. | VIM direct correct hits + VIM direct correct passes + VIM delayed correct hits + VIM delayed correct passes. | Visual memory (direct and delayed) |
| Finger tapping Test (FTT) | Subject presses space bar as many times as possible in 10 seconds with the index finger (three trials per side). | FTT average taps right + FTT average taps left. | Motor Speed |
| _a_Non-computerized test | | | |

## Supplementary Table 2

| Supplementary Table 2: Univariable linear regression model demonstrating correlation and linear coefficients between cognitive test *z* scores and the coordinate on the anterior-posterior axis. Positive correlations indicate better performances with more frontal coordinates. | | | | | | | |
| --- | --- | --- | --- | --- | --- | --- | --- |
|  | **Correlation** | | | | **Univariate linear regression** | | |
|  | *No. of patients* | *Pearson’s R* | *R^2^* | *p value* | *Beta_a_* | *F test* | *p value* |
| *Shifting Attention Test* | 309 | -.020 | <.001 | .363 | -0.011 | 0.123 | .726 |
| *Stroop Test Interference* | 306 | -.118 | .014 | **.019*** | -0.045 | 4.323 | **.038*** |
| *Verbal Fluency* | 241 | -.103 | .103 | .055 | -0.028 | 2.560 | .111 |
| *Digit Span Backward* | 135 | .047 | .002 | .295 | 0.012 | 0.291 | .591 |
| *Continuous Performance Test* | 312 | -.071 | .005 | .105 | -0.026 | 1.580 | .210 |
| *Digit Span Forward* | 135 | -.030 | .001 | .367 | -0.009 | 0.117 | .733 |
| *Symbol Digit Coding* | 320 | .113 | .013 | **.022*** | 0.041 | 4.113 | **.043*** |
| *Stroop Test part 1* | 315 | .065 | .004 | .125 | 0.038 | 1.155 | .249 |
| *Verbal Memory test* | 315 | .016 | <.001 | .387 | 0.006 | 0.082 | .775 |
| *Visual Memory test* | 313 | .060 | .004 | .144 | 0.019 | 1.130 | .289 |
| *Finger Tapping test* | 317 | .028 | .001 | .313 | 0.012 | 0.239 | .626 |
| Statistical significance was considered as *p*<0.05, marked by the asterisk in bold. _a_Beta coefficient, indicating the increase in cognitive *z* score per centimeter increase in frontal coordinate. Positive correlations/Beta values indicate better performances with more frontal coordinates. | | | | | | | |

## Supplementary Table 3

| Supplementary Table 3 (1/3): Multivariable logistic regression analyses for predicting impairment on each EF test in meningioma patients. | | | | | | | | |
| --- | --- | --- | --- | --- | --- | --- | --- | --- |
|  | **Shifting Attention Test** | | **Stroop Test Interference Ratio** | | **Verbal Fluency** | | **Digit Span Backward** | |
| *N (N impaired)* | 225 (82) | | 220 (27) | | 178 (52) | | 135 (32) | |
| *Nagelkerke R^2^_a_* | .198 | | .147 | | .266 | | .307 | |
| *Classification accuracy (%)_b_* | 69.3% | | 87.7% | | 74.7% | | 78.5% | |
| *Model p value_c_* | **.002*** | | .278 | | **.001*** | | **.002*** | |
|  | *OR (95% CI)_d_* | *p value* | *OR (95% CI)_d_* | *p value* | *OR (95% CI)_d_* | *p value* | *OR (95% CI)_d_* | *p value* |
| *Frontal coordinate (posterior to anterior) (cm)* | 1.01 (0.93 – 1.10) | .786 | 1.06 (0.93 – 1.21) | .365 | 1.09 (0.98 – 1.21) | .113 | 0.92 (0.81 – 1.05) | .233 |
| *Height coordinate (caudocranial) (cm)* | 1.01 (0.91 – 1.11) | .906 | 1.02 (0.88 – 1.18) | .789 | 0.95 (0.84 – 1.07) | .393 | 1.05 (0.89 – 1.25) | .571 |
| *Frontal * Height interaction* | 1.01 (0.98 – 1.04) | .518 | 0.98 (0.94 – 1.03) | .462 | 1.04 (1.00 – 1.08) | .079 | 0.97 (0.93 – 1.02) | .296 |
| *Lateral coordinate (left to right) (cm)* | 0.90 (0.81 – 1.01) | .070 | 0.80 (0.68 – 0.94) | .007 | **0.80 (0.69 – 0.92)** | **.002*** | 0.83 (0.70 – 0.99) | .036 |
| *Frontal * Lateral interaction* | 1.01 (0.97 – 1.04) | .698 | 0.96 (0.91 – 1.01) | .138 | 0.97 (0.92 – 1.01) | .108 | 1.02 (0.96 – 1.08) | .483 |
| *Tumor volume (cm^3^)* | **1.02 (1.01 – 1.02)** | **<.001*** | 1.01 (1.00 – 1.02) | .123 | 1.01 (1.00 – 1.03) | .011 | 1.00 (0.99 – 1.02) | .526 |
| *Frontal * Volume interaction* | 1.00 (1.00 – 1.00) | .429 | 1.00 (1.00 – 1.00) | .660 | 1.00 (1.00 – 1.00) | .816 | 1.00 (1.00 – 1.00) | .810 |
| *Age (years)* | 1.03 (1.00 – 1.06) | .088 | 1.04 (0.99 – 1.07) | .177 | 1.01 (0.97 – 1.05) | .715 | **0.92 (0.88 – 0.97)** | **.001*** |
| *Female sex (vs. male)* | 0.94 (0.49 – 1.84) | .863 | 0.71 (0.29 – 1.77) | .461 | 0.68 (0.30 – 1.52) | .347 | 1.58 (0.53 – 4.66) | .411 |
| *Educational level_e_* |  |  |  |  |  |  |  |  |
| *Middle (vs. low)* | 0.68 (0.32 – 1.43) | .308 | 1.34 (0.46 – 3.90) | .592 | 0.37 (0.14 – 0.97) | .044 | 1.40 (0.33 – 5.95) | .650 |
| *High (vs. low)* | 0.73 (0.33 – 1.58) | .419 | 0.75 (0.23 – 2.41) | .627 | 0.31 (0.11 – 0.84) | .022 | 1.22 (0.30 – 4.94) | .786 |
| *WHO Grade II (vs. Grade I)* | 1.73 (0.55 – 5.46) | .351 | 0.37 (0.04 – 3.30) | .374 | 1.32 (0.35 – 5.04) | .681 | 3.09 (0.69 – 13.75) | .139 |
| *ASA grade III/IV (vs. grade I/II)* | 0.80 (0.35 – 1.84) | .595 | 0.68 (0.20 – 2.26) | .524 | 1.50 (0.56 – 4.00) | .420 |  |  |
| *HADS anxiety (points)_f_* | 1.00 (0.92 – 1.08) | .879 | 0.96 (0.85 – 1.09) | .540 | 0.97 (0.88 – 1.08) | .569 |  |  |
| *HADS depression (points)_f_* | 1.07 (0.99 – 1.16) | .085 | 0.98 (0.88 – 1.10) | .778 | 1.02 (0.93 – 1.13) | .640 |  |  |
|  | p<.003 | | p<.003 | | p<.003 | | p<.004 | |
| The number of patients differs over domains due to missing or invalid scores. Alpha was adjusted using the Benjamini-Hochberg procedure. Statistical significance per test depends on the number of covariates and is marked in bold. _a_Nagelkerke R^2^, larger R^2^ values indicate more variance explained by the model, to a maximum of 1. _b_Percentage Accuracy in Classification (PAC), the percentage of the total sample that is correctly classified by the model. _c_Statistical significance was considered as *p*<0.05. _d_Odds ratios (OR) for the predictors, ORs larger than 1.00 indicate an increased odd of impairment for a one unit increase of the first-listed predictor variable. _e_According to the Dutch Verhage scale. _f_HADS, Hospital Anxiety and Depression Scale. | | | | | | | | |

| Supplementary Table 3 (2/3): Multivariable logistic regression analyses for predicting impairment on non-EF tests in meningioma patients. | | | | | | | | |
| --- | --- | --- | --- | --- | --- | --- | --- | --- |
|  | **Continuous Performance Test** | | **Digit Span Forward** | | **Symbol Digit Coding** | | **Stroop Test Part 1** | |
| *N (N impaired)* | 225 (37) | | 135 (18) | | 233 (79) | | 227 (58) | |
| *Nagelkerke R^2^_a_* | .211 | | .446 | | .188 | | .197 | |
| *Classification accuracy (%)_b_* | 82.7% | | 88.1% | | 70.8% | | 77.5% | |
| *Model p value_c_* | **.012*** | | **<.001*** | | **.003*** | | **.005*** | |
|  | *OR (95% CI)_d_* | *p value* | *OR (95% CI)_d_* | *p value* | *OR (95% CI)_d_* | *p value* | *OR (95% CI)_d_* | *p value* |
| *Frontal coordinate (posterior to anterior) (cm)* | 1.17 (1.03 – 1.32) | .017 | 0.90 (0.74 – 1.08) | .248 | 0.98 (0.90 – 1.06) | .584 | 1.03 (0.94 – 1.13) | .590 |
| *Height coordinate (caudocranial) (cm)* | 0.96 (0.84 – 1.10) | .597 | 1.20 (0.93 – 1.54) | .171 | 0.99 (0.90 – 1.09) | .818 | 1.03 (0.93 – 1.15) | .596 |
| *Frontal * Height interaction* | 0.99 (0.94 – 1.03) | .565 | 1.00 (0.93 – 1.07) | .986 | 0.99 (0.96 – 1.02) | .409 | 1.01 (0.97 – 1.04) | .696 |
| *Lateral coordinate (left to right) (cm)* | 0.94 (0.81 – 1.08) | .374 | 1.32 (1.02 – 1.71) | .038 | 1.00 (0.90 – 1.12) | .995 | 1.00 (0.89 – 1.13) | .963 |
| *Frontal * Lateral interaction* | 0.96 (0.92 – 1.01) | .134 | 1.04 (0.96 – 1.13) | .301 | 0.98 (0.95 – 1.01) | .264 | 0.99 (0.95 – 1.02) | .493 |
| *Tumor volume (cm^3^)* | 1.01 (1.00 – 1.02) | .133 | 1.00 (0.98 – 1.02) | .911 | **1.01 (1.01 – 1.02)** | **.001*** | 1.00 (0.99 – 1.01) | .707 |
| *Frontal * Volume interaction* | 1.00 (1.00 – 1.00) | .839 | 1.00 (1.00 – 1.00) | .693 | 1.00 (1.00 – 1.00) | .369 | 1.00 (1.00 – 1.00) | .205 |
| *Age (years)* | 1.01 (0.98 – 1.05) | .559 | **0.89 (0.83 – 0.96)** | **.001*** | 1.02 (1.00 – 1.05) | .108 | **1.06 (1.02 – 1.09)** | **.002*** |
| *Female sex (vs. male)* | 1.47 (0.58 – 3.72) | .415 | 9.46 (1.54 – 58.08) | .015 | 1.68 (0.85 – 3.32) | .133 | 0.66 (0.32 – 1.36) | .261 |
| *Educational level_e_* |  |  |  |  |  |  |  |  |
| *Middle (vs. low)* | 0.53 (0.21 – 1.33) | .175 | 0.50 (0.04 – 6.31) | .593 | 0.73 (0.35 – 1.53) | .404 | 0.55 (0.24 – 1.25) | .156 |
| *High (vs. low)* | 0.21 (0.07 – 0.66) | .007 | 3.81 (0.46 – 31.88) | .217 | 0.57 (0.26 – 1.24) | .156 | 0.58 (0.26 – 1.42) | .246 |
| *WHO Grade II (vs. Grade I)* | 0.22 (0.02 – 2.42) | .215 | 3.35 (0.45 – 24.86) | .236 | 0.82 (0.25 – 2.74) | .751 | 0.58 (0.12 – 2.85) | .503 |
| *ASA grade III/IV (vs. grade I/II)* | 0.75 (0.26 – 2.18) | .599 |  |  | 0.95 (0.41 – 2.19) | .897 | 0.63 (0.25 – 1.61) | .332 |
| *HADS anxiety (points)_f_* | 0.98 (0.89 – 1.09) | .738 |  |  | 1.11 (1.02 – 1.20) | .013 | 1.06 (0.97 – 1.16) | .192 |
| *HADS depression (points)_f_* | 1.11 (1.01 – 1.22) | .037 |  |  | 0.99 (0.91 – 1.07) | .721 | 1.05 (0.96 – 1.15) | .284 |
|  | p<.003 | | p<.004 | | p<.003 | | p<.003 | |
| The number of patients differs over domains due to missing or invalid scores. Alpha was adjusted using the Benjamini-Hochberg procedure. Statistical significance per test depends on the number of covariates and is marked in bold. _a_Nagelkerke R^2^, larger R^2^ values indicate more variance explained by the model, to a maximum of 1. _b_Percentage Accuracy in Classification (PAC), the percentage of the total sample that is correctly classified by the model. _c_Statistical significance was considered as *p*<0.05. _d_Odds ratios (OR) for the predictors, ORs larger than 1.00 indicate an increased odd of impairment for a one unit increase of the first-listed predictor variable. _e_According to the Dutch Verhage scale. _f_HADS, Hospital Anxiety and Depression Scale. | | | | | | | | |

| Supplementary Table 3 (3/3): Multivariable logistic regression analyses for predicting impairment on non-EF tests in meningioma patients. | | | | | | |
| --- | --- | --- | --- | --- | --- | --- |
|  | **Verbal Memory** | | **Visual Memory** | | **Finger Tapping Test** | |
| *N (N impaired)* | 227 (58) | | 225 (44) | | 212 (78) | |
| *Nagelkerke R^2^_a_* | .128 | | .138 | | .221 | |
| *Classification accuracy (%)_b_* | 75.3% | | 80.4% | | 73.6% | |
| *Model p value_c_* | .146 | | .138 | | **<.001*** | |
|  | *OR (95% CI)_d_* | *p value* | *OR (95% CI)_d_* | *p value* | *OR (95% CI)_d_* | *p value* |
| *Frontal coordinate (posterior to anterior) (cm)* | 1.00 (0.92 – 1.09) | .969 | 0.89 (0.80 – 0.98) | .020 | 1.01 (0.93 – 1.10) | .757 |
| *Height coordinate (caudocranial) (cm)* | 0.99 (0.88 – 1.10) | .782 | 0.98 (0.86 – 1.11) | .711 | 0.98 (0.88 – 1.08) | .646 |
| *Frontal * Height interaction* | 1.03 (0.99 – 1.06) | .118 | 0.98 (0.94 – 1.01) | .220 | 1.00 (0.97 – 1.03) | .841 |
| *Lateral coordinate (left to right) (cm)* | 1.08 (0.96 – 1.21) | .221 | 1.03 (0.90 – 1.18) | .634 | 1.00 (0.90 – 1.13) | .948 |
| *Frontal * Lateral interaction* | 0.98 (0.94 – 1.01) | .211 | 0.99 (0.96 – 1.03) | .707 | 1.00 (0.96 – 1.03) | .920 |
| *Tumor volume (cm^3^)* | 1.00 (0.99 – 1.01) | .705 | 1.01 (1.00 – 1.02) | .022 | 1.01 (1.00 – 1.02) | .092 |
| *Frontal * Volume interaction* | 1.00 (1.00 – 1.00) | .212 | 1.00 (1.00 – 1.00) | .201 | 1.00 (1.00 – 1.00) | .571 |
| *Age (years)* | 1.01 (0.98 – 1.04) | .633 | 1.01 (0.97 – 1.04) | .718 | **1.05 (1.02 – 1.08)** | **.002*** |
| *Female sex (vs. male)* | 0.90 (0.45 – 1.81) | .775 | 0.89 (0.41 – 1.92) | .765 | 1.18 (0.60 – 2.34) | .631 |
| *Educational level_e_* |  |  |  |  |  |  |
| *Middle (vs. low)* | 0.73 (0.34 – 1.60) | .435 | 0.97 (0.40 – 2.32) | .938 | 0.72 (0.35 – 1.48) | .374 |
| *High (vs. low)* | 0.52 (0.23 – 1.19) | .122 | 0.74 (0.29 – 1.86) | .517 | 0.34 (0.15 – 0.77) | .010 |
| *WHO Grade II (vs. Grade I)* | 0.48 (0.09 – 2.42) | .371 | 0.54 (0.10 – 3.02) | .484 | 1.28 (0.40 – 4.10) | .682 |
| *ASA grade III/IV (vs. grade I/II)* | 0.40 (0.14 – 1.16) | .091 | 0.44 (0.14 – 1.39) | .162 | 1.01 (0.44 – 2.28) | .990 |
| *HADS anxiety (points)_f_* | 0.92 (0.84 – 1.00) | .057 | 1.00 (0.92 – 1.10) | .934 | 1.03 (0.95 – 1.12) | .478 |
| *HADS depression (points)_f_* | 1.09 (1.01 – 1.19) | .035 | 1.07 (0.98 – 1.17) | .142 | 1.04 (0.96 – 1.13) | .360 |
|  | p<.003 | | p<.003 | | p<.003 | |
| The number of patients differs over domains due to missing or invalid scores. Alpha was adjusted using the Benjamini-Hochberg procedure. Statistical significance per test depends on the number of covariates and is marked in bold. _a_Nagelkerke R^2^, larger R^2^ values indicate more variance explained by the model, to a maximum of 1. _b_Percentage Accuracy in Classification (PAC), the percentage of the total sample that is correctly classified by the model. _c_Statistical significance was considered as *p*<0.05. _d_Odds ratios (OR) for the predictors, ORs larger than 1.00 indicate an increased odd of impairment for a one unit increase of the first-listed predictor variable. _e_According to the Dutch Verhage scale. _f_HADS, Hospital Anxiety and Depression Scale. | | | | | | |

## Supplementary Table 4

| Supplementary Table 4: Multivariable logistic regression analyses for digit span, excluding age as a covariate, predicting impairment in meningioma patients. | | | | |
| --- | --- | --- | --- | --- |
|  | **Digit Span Backward** | | **Digit Span Forward** | |
| *N (N impaired)* | 135 (32) | | 135 (18) | |
| *Nagelkerke R^2^_a_* | .185 | | .289 | |
| *Classification accuracy (%)_b_* | 74.8% | | 88.9% | |
| *Model p value_c_* | .087 | | **.017*** | |
|  | *OR (95% CI)_d_* | *p value* | *OR (95% CI)_d_* | *p value* |
| *Frontal coordinate (posterior to anterior) (cm)* | 0.93 (0.83 – 1.05) | .252 | 0.92 (0.78 – 1.08) | .300 |
| *Height coordinate (caudocranial) (cm)* | 1.02 (0.87 – 1.19) | .841 | 1.10 (0.90 – 1.36) | .359 |
| *Frontal * Height interaction* | 0.97 (0.92 – 1.01) | .159 | 0.99 (0.93 – 1.05) | .677 |
| *Lateral coordinate (left to right) (cm)* | 0.83 (0.71 – 0.98) | .026 | 1.18 (0.95 – 1.46) | .134 |
| *Frontal * Lateral interaction* | 1.01 (0.95 – 1.06) | .838 | 1.02 (0.95 – 1.09) | .676 |
| *Tumor volume (cm^3^)* | 1.00 (0.99 – 1.02) | .580 | 1.00 (0.99 – 1.02) | .992 |
| *Frontal * Volume interaction* | 1.00 (0.99 – 1.01) | .545 | 1.00 (1.00 – 1.01) | .534 |
| *Female sex (vs. male)* | 1.62 (0.59 – 4.47) | .351 | 8.16 (1.39 – 47.79) | .020 |
| *Educational level_e_* |  |  |  |  |
| *Middle (vs. low)* | 2.52 (0.68 – 9.28) | .165 | 1.30 (0.16 – 10.85) | .812 |
| *High (vs. low)* | 2.58 (0.75 – 8.87) | .133 | 7.88 (1.22 – 50.86) | .030 |
| *WHO Grade II (vs. Grade I)* | 3.24 (0.77 – 13.75) | .110 | 3.37 (0.56 – 20.38) | .185 |
|  | p<.005 | | p<.005 | |
| The number of patients differs over domains due to missing or invalid scores. Alpha was adjusted using the Benjamini-Hochberg procedure. Statistical significance per test depends on the number of covariates and is marked in bold. _a_Nagelkerke R^2^, larger R^2^ values indicate more variance explained by the model, to a maximum of 1. _b_Percentage Accuracy in Classification (PAC), the percentage of the total sample that is correctly classified by the model. _c_Statistical significance was considered as *p*<0.05. _d_Odds ratios (OR) for the predictors, ORs larger than 1.00 indicate an increased odd of impairment for a one unit increase of the first-listed predictor variable. _e_According to the Dutch Verhage scale. | | | | |

## Supplementary Table 5

| Supplementary Table 5 (1/3): Multivariable logistic regression analyses for predicting impairment on each EF test in meningioma patients. Frontal lobe based anatomical labels. | | | | | | | | |
| --- | --- | --- | --- | --- | --- | --- | --- | --- |
|  | **Shifting Attention Test** | | **Stroop Test Interference Ratio** | | **Verbal Fluency** | | **Digit Span Backward** | |
| *N (N impaired)* | 22 (81) | | 219 (26) | | 177 (52) | | 133 (31) | |
| *Nagelkerke R^2^_a_* | .279 | | .133 | | .239 | | .314 | |
| *Classification accuracy (%)_b_* | 74.6% | | 88.6% | | 74.6% | | 77.4% | |
| *Model p value_c_* | **<.001*** | | .550 | | **.013*** | | **.006*** | |
|  | *OR (95% CI)_d_* | *p value* | *OR (95% CI)_d_* | *p value* | *OR (95% CI)_d_* | *p value* | *OR (95% CI)_d_* | *p value* |
| *Frontal localization*  *Solely Frontal (vs. non-frontal)*  *Frontally Involved (vs. non-frontal)* | 0.90 (0.34 – 2.41)  2.70 (0.77 – 9.48) | .840  .121 | 0.75 (0.20 – 2.84)  0.14 (0.01 – 3.10) | .677  .212 | 0.70 (0.21 – 2.38)  0.99 (0.21 – 4.72) | .571  .994 | 0.25 (0.03 – 2.51)  1.23 (0.21 – 7.16) | .240  .815 |
| *Hemisphere_e_* |  |  |  |  |  |  |  |  |
| *Left (vs. right)* | 1.80 (0.66 – 4.91) | .254 | 1.86 (0.50 – 6.99) | .358 | 2.92 (0.85 – 10.07) | .090 | 3.02 (0.71 – 12.89) | .135 |
| *Bilateral (vs. right)* | 0.85 (0.27 – 2.67) | .777 | 2.60 (0.66 – 10.33) | .174 | 3.06 (0.82 – 11.37) | .096 | 1.25 (0.11 – 14.48) | .861 |
| *Fr. Lobe * Left interaction* | 1.89 (0.44 – 8.19) | .394 | 1.40 (0.21 – 9.47) | .728 | 1.03 (0.17 – 6.12) | .977 | 1.50 (0.09 – 25.03) | .779 |
| *Fr. Involved * Left Interaction* | 1.07 (0.17 – 6.55) | .944 | 0.54 (0.02 – 19.78) | .735 | 2.69 (0.33 – 21.91) | .355 | 0.73 (0.07 – 7.87) | .793 |
| *Fr. Lobe * Bilateral interaction* | - | - | - | - | - | - | - | - |
| *Tumor volume (cm^3^)* | 1.01 (0.99 – 1.02) | .346 | 1.01 (0.99 – 1.03) | .327 | 1.00 (0.99 – 1.02) | .635 | 1.00 (0.97 – 1.02) | .825 |
| *Fr. Lobe * Volume interaction*  *Fr. Involved * Volume Interaction* | 1.01 (0.99 – 1.02)  1.03 (1.00 – 1.05) | .593  .082 | 1.00 (0.98 – 1.02)  1.03 (0.98 – 1.09) | .825  .261 | 1.01 (0.99 – 1.04)  1.01 (0.98 – 1.04) | .244  .558 | 1.02 (0.98 – 1.05)  1.01 (0.98 – 1.04) | .387  .704 |
| *Age (years)* | 1.04 (1.00 – 1.07) | .028 | 1.03 (0.98 – 1.07) | .236 | 1.01 (0.98 – 1.05) | .442 | **0.93 (0.88 – 0.97)** | **.002*** |
| *Female sex (vs. male)* | 0.89 (0.45 – 1.79) | .752 | 0.74 (0.29 – 1.84) | .511 | 0.59 (0.26 – 1.31) | .195 | 1.13 (0.37 – 3.43) | .828 |
| *Educational level_f_* |  |  |  |  |  |  |  |  |
| *Middle (vs. low)* | 0.52 (0.24 – 1.14) | .103 | 1.40 (0.48 – 4.09) | .545 | 0.38 (0.15 – 0.98) | .046 | 1.14 (0.27 – 4.90) | .858 |
| *High (vs. low)* | 0.60 (0.26 – 1.37) | .223 | 1.01 (0.31 – 3.27) | .983 | 0.31 (0.12 – 0.82) | .019 | 1.13 (0.29 – 4.49) | .860 |
| *WHO Grade II (vs. Grade I)* | 1.77 (0.54 – 5.80) | .349 | 0.50 (0.06 – 4.38) | .527 | 1.17 (0.31 – 4.45) | .814 | 4.69 (1.06 – 20.67) | .042 |
| *ASA grade III/IV (vs. grade I/II)* | 0.81 (0.34 – 1.95) | .641 | 0.76 (0.23 – 2.54) | .652 | 1.52 (0.57 – 4.02) | .404 |  |  |
| *HADS anxiety (points)_g_* | 0.97 (0.89 – 1.06) | .485 | 0.95 (0.84 – 1.07) | .383 | 0.96 (0.87 – 1.07) | .506 |  |  |
| *HADS depression (points)_g_* | 1.10 (1.01 – 1.19) | .032 | 1.03 (0.92 – 1.15) | .640 | 1.03 (0.93 – 1.13) | .560 |  |  |
|  | p<.003 | | p<.003 | | p<.003 | | p<.004 | |
| The number of patients differs over domains due to missing or invalid scores. Alpha was adjusted using the Benjamini-Hochberg procedure. Statistical significance per test depends on the number of covariates and is marked in bold. _a_Nagelkerke R^2^, larger R^2^ values indicate more variance explained by the model, to a maximum of 1. _b_Percentage Accuracy in Classification (PAC), the percentage of the total sample that is correctly classified by the model. _c_Statistical significance was considered as *p*<0.05. _d_Odds ratios (OR) for the predictors, ORs larger than 1.00 indicate an increased odd of impairment for a one unit increase of the first-listed predictor variable. _e_No interaction effect between frontally involved meningiomas and bilateral meningiomas was computed, as no such patients were present. Accordingly, no interaction effect between solely frontal and bilateral meningiomas was computed, if no such patients were present. _f_According to the Dutch Verhage scale. _g_HADS, Hospital Anxiety and Depression Scale. | | | | | | | | |

| Supplementary Table 5 (2/3): Multivariable logistic regression analyses for predicting impairment on non-EF tests in meningioma patients. Frontal lobe based anatomical labels. | | | | | | | | |
| --- | --- | --- | --- | --- | --- | --- | --- | --- |
|  | **Continuous Performance Test** | | **Digit Span Forward** | | **Symbol Digit Coding** | | **Stroop Test Part 1** | |
| *N (N impaired)* | 224 (36) | | 133 (18) | | 232 (78) | | 226 (58) | |
| *Nagelkerke R^2^_a_* | .232 | | .432 | | .220 | | .219 | |
| *Classification accuracy (%)_b_* | 82.1% | | 86.5% | | 72.0% | | 79.2% | |
| *Model p value_c_* | **.012*** | | **<.001*** | | **.001*** | | **.004*** | |
|  | *OR (95% CI)_d_* | *p value* | *OR (95% CI)_d_* | *p value* | *OR (95% CI)_d_* | *p value* | *OR (95% CI)_d_* | *p value* |
| *Frontal localization*  *Solely Frontal (vs. non-frontal)*  *Frontally Involved (vs. non-frontal)* | 1.97 (0.57 – 6.86)  1.78 (0.36 – 8.79) | .286  .481 | 0.22 (0.02 – 2.75)  3.48 (0.51 – 23.90) | .238  .205 | 0.35 (0.14 – 0.91)  0.64 (0.20 – 2.02) | .030  .448 | 1.52 (0.54 – 4.27)  2.07 (0.58 – 7.36) | .429  .263 |
| *Hemisphere_e_* |  |  |  |  |  |  |  |  |
| *Left (vs. right)* | 0.76 (0.16 – 3.67) | .735 | 1.83 (0.27 – 12.48) | .538 | 0.40 (0.14 – 1.09) | .073 | 1.03 (0.32 – 3.34) | .958 |
| *Bilateral (vs. right)* | 3.08 (0.92 – 10.28) | .068 |  | - | 2.56 (0.90 – 7.29) | .078 | 1.32 (0.44 – 4.02) | .621 |
| *Fr. Lobe * Left interaction* | 2.55 (0.35 – 18.61) | .356 | 1.30 (0.04 – 44.93) | .884 | 3.65 (0.79 – 16.85) | .097 | 1.13 (0.22 – 5.69) | .883 |
| *Fr. Involved * Left Interaction* | 1.04 (0.09 – 12.31) | .977 | 0.11 (0.01 – 2.13) | .144 | 4.15 (0.76 – 22.53) | .099 | 2.11 (0.34 – 13.20) | .427 |
| *Fr. Lobe * Bilateral interaction* | - | - |  | - | - | - | - | - |
| *Tumor volume (cm^3^)* | 1.01 (0.98 – 1.03) | .666 | 0.99 (0.95 – 1.02) | .432 | 1.02 (1.01 – 1.04) | .005 | 1.00 (0.98 – 1.02) | .833 |
| *Fr. Lobe * Volume interaction*  *Fr. Involved * Volume Interaction* | 1.01 (0.98 – 1.03)  1.01 (0.97 – 1.04) | .580  .765 | 1.02 (0.97 – 1.08)  1.01 (0.97 – 1.05) | .373  .534 | 0.99 (0.97 – 1.01)  0.98 (0.96 – 1.01) | .366  .124 | 1.00 (0.98 – 1.02)  1.01 (0.98 – 1.03) | .993  .599 |
| *Age (years)* | 1.00 (0.97 – 1.04) | .901 | **0.90 (0.84 – 0.96)** | **.002*** | 1.02 (1.00 – 1.05) | .108 | **1.06 (1.02 – 1.09)** | **.001*** |
| *Female sex (vs. male)* | 1.36 (0.54 – 3.46) | .516 | 7.44 (1.10 – 50.27) | .039 | 1.75 (0.86 – 3.53) | .121 | 0.63 (0.30 – 1.30) | .211 |
| *Educational level_f_* |  |  |  |  |  |  |  |  |
| *Middle (vs. low)* | 0.54 (0.21 – 1.40) | .201 | 0.66 (0.06 – 7.31) | .731 | 0.70 (0.33 – 1.49) | .357 | 0.47 (0.20 – 1.07) | .073 |
| *High (vs. low)* | 0.22 (0.07 – 0.69) | .009 | 3.97 (0.44 – 35.97) | .220 | 0.52 (0.23 – 1.17) | .111 | 0.48 (0.20 – 1.17) | .106 |
| *WHO Grade II (vs. Grade I)* | 0.21 (0.02 – 2.30) | .199 | 9.45 (1.19 – 75.55) | .034 | 0.83 (0.25 – 2.77) | .757 | 0.54 (0.11 – 2.65) | .447 |
| *ASA grade III/IV (vs. grade I/II)* | 0.67 (0.22 – 2.05) | .485 |  |  | 1.11 (0.48 – 2.60) | .804 | 0.62 (0.24 – 1.58) | .313 |
| *HADS anxiety (points)_g_* | 0.99 (0.89 – 1.10) | .783 |  |  | 1.12 (1.02 – 1.22) | .014 | 1.06 (0.96 – 1.16) | .235 |
| *HADS depression (points)_g_* | 1.13 (1.02 – 1.25) | .020 |  |  | 0.98 (0.90 – 1.06) | .595 | 1.04 (0.95 – 1.14) | 388 |
|  | p<.003 | | p<.004 | | p<.003 | | p<.003 | |
| The number of patients differs over domains due to missing or invalid scores. Alpha was adjusted using the Benjamini-Hochberg procedure. Statistical significance per test depends on the number of covariates and is marked in bold. _a_Nagelkerke R^2^, larger R^2^ values indicate more variance explained by the model, to a maximum of 1. _b_Percentage Accuracy in Classification (PAC), the percentage of the total sample that is correctly classified by the model. _c_Statistical significance was considered as *p*<0.05. _d_Odds ratios (OR) for the predictors, ORs larger than 1.00 indicate an increased odd of impairment for a one unit increase of the first-listed predictor variable. _e_No interaction effect between frontally involved meningiomas and bilateral meningiomas was computed, as no such patients were present. Accordingly, no interaction effect between solely frontal and bilateral meningiomas was computed, if no such patients were present. _f_According to the Dutch Verhage scale. _g_HADS, Hospital Anxiety and Depression Scale. | | | | | | | | |

| Supplementary Table 5 (3/3): Multivariable logistic regression analyses for predicting impairment on non-EF tests in meningioma patients. Frontal lobe based anatomical labels. | | | | | | |
| --- | --- | --- | --- | --- | --- | --- |
|  | **Verbal Memory** | | **Visual Memory** | | **Finger Tapping Test** | |
| *N (N impaired)* | 226 (58) | | 224 (43) | | 230 (78) | |
| *Nagelkerke R^2^_a_* | .180 | | .141 | | .230 | |
| *Classification accuracy (%)_b_* | 75.2% | | 79.5% | | 73.0% | |
| *Model p value_c_* | .042 | | .242 | | **<.001*** | |
|  | *OR (95% CI)_d_* | *p value* | *OR (95% CI)_d_* | *p value* | *OR (95% CI)_d_* | *p value* |
| *Frontal localization*  *Solely Frontal (vs. non-frontal)*  *Frontally Involved (vs. non-frontal)* | 0.76 (0.28 – 2.10)  0.59 (0.15 – 2.27) | .599  .441 | 0.32 (0.11 – 0.93)  0.70 (0.19 – 2.49) | .037  .579 | 1.00 (0.40 – 2.49)  2.21 (0.68 – 7.12) | .998  .186 |
| *Hemisphere_e_* |  |  |  |  |  |  |
| *Left (vs. right)* | 0.64 (0.22 – 1.84) | .404 | 0.49 (0.16 – 1.51) | .216 | 1.04 (0.39 – 2.79) | .945 |
| *Bilateral (vs. right)* | 0.51 (0.05 – 5.66) | .580 | 1.68 (0.47 – 5.97) | .423 | 1.33 (0.48 – 3.70) | .590 |
| *Fr. Lobe * Left interaction* | 1.14 (0.22 – 5.85) | .875 | 2.14 (0.36 – 12.79) | .403 | 0.89 (0.21 – 3.84) | .872 |
| *Fr. Involved * Left Interaction* | 2.24 (0.34 – 14.94) | .406 | 1.79 (0.26 – 12.31) | .553 | 0.43 (0.07 – 2.61) | .358 |
| *Fr. Lobe * Bilateral interaction* | 1.29 (0.09 – 19.13) | .855 | - | - | - | - |
| *Tumor volume (cm^3^)* | 1.00 (0.99 – 1.02) | .916 | 1.01 (0.99 – 1.02) | .404 | 1.01 (0.99 – 1.02) | .383 |
| *Fr. Lobe * Volume interaction*  *Fr. Involved * Volume Interaction* | 0.97 (0.95 – 1.00)  1.03 (1.00 – 1.05) | .029  .079 | 1.01 (0.99 – 1.03)  1.01 (0.99 – 1.04) | .461  .406 | 1.00 (0.98 – 1.02)  0.99 (0.97 – 1.02) | .913  .524 |
| *Age (years)* | 1.02 (0.99 – 1.05) | .280 | 1.01 (0.98 – 1.04) | .541 | **1.05 (1.02 – 1.08)** | **.002*** |
| *Female sex (vs. male)* | 0.93 (0.45 – 1.91) | .832 | 0.80 (0.37 – 1.76) | .586 | 1.16 (0.58 – 2.32) | .670 |
| *Educational level_f_* |  |  |  |  |  |  |
| *Middle (vs. low)* | 0.67 (0.31 – 1.46) | .311 | 0.84 (0.34 – 2.06) | .698 | 0.77 (0.37 – 1.58) | .475 |
| *High (vs. low)* | 0.41 (0.17 – 0.98) | .044 | 0.74 (0.29 – 1.90) | .531 | 0.34 (0.15 – 0.78) | .011 |
| *WHO Grade II (vs. Grade I)* | 0.41 (0.08 – 2.09) | .284 | 0.72 (0.14 – 3.80) | .702 | 1.34 (0.42 – 4.27) | .616 |
| *ASA grade III/IV (vs. grade I/II)* | 0.28 (0.09 – 0.86) | .027 | 0.52 (0.17 – 1.62) | .258 | 1.06 (0.46 – 2.43) | .897 |
| *HADS anxiety (points)_g_* | 0.91 (0.83 – 1.00) | .046 | 1.01 (0.91 – 1.11) | .855 | 1.03 (0.95 – 1.12) | .496 |
| *HADS depression (points)_g_* | 1.11 (1.02 – 1.21) | .019 | (1.07 (0.98 – 1.17) | .134 | 1.04 (0.96 – 1.13) | .336 |
|  | p<.003 | | p<.003 | | p<.003 | |
| The number of patients differs over domains due to missing or invalid scores. Alpha was adjusted using the Benjamini-Hochberg procedure. Statistical significance per test depends on the number of covariates and is marked in bold. _a_Nagelkerke R^2^, larger R^2^ values indicate more variance explained by the model, to a maximum of 1. _b_Percentage Accuracy in Classification (PAC), the percentage of the total sample that is correctly classified by the model. _c_Statistical significance was considered as *p*<0.05. _d_Odds ratios (OR) for the predictors, ORs larger than 1.00 indicate an increased odd of impairment for a one unit increase of the first-listed predictor variable. _e_No interaction effect between frontally involved meningiomas and bilateral meningiomas was computed, as no such patients were present. Accordingly, no interaction effect between solely frontal and bilateral meningiomas was computed, if no such patients were present. _f_According to the Dutch Verhage scale. _g_HADS, Hospital Anxiety and Depression Scale. | | | | | | |
